# Iron Depletion: An Ameliorating Factor for Sickle Cell Disease?

**DOI:** 10.5402/2011/473152

**Published:** 2011-07-05

**Authors:** P. C. Giordano, W. Huisman, C. L. Harteveld

**Affiliations:** ^1^Hemoglobinopathies Laboratory, Department of Human and Clinical Genetics, Leiden University Medical Center, Einthovenweg 20, 2300RC Leiden, The Netherlands; ^2^Department of Clinical Chemistry, Westeinde Hospital, Lijnbaan 32, 2512 VA, The Hague, The Netherlands

## Abstract

We report some observations from our laboratory practice that might be important for the treatment of sickle cell disease (SCD). We describe data from two cases indicating that iron depletion might have a beneficial effect diminishing the formation of HbS in favor of HbF, possibly reducing the severity of the disease. We believe that it would be worthwhile to monitor the course of the disease comparing cases with identical genotypes with and without iron depletion, and we advise to consider chelation therapy to reduce iron overload in patients with SCD.

## 1. Introduction

Sickle cell disease (SCD), also called sickle cell anemia (SCA), is one of the most common recessive conditions in human kind. Selected by malaria, carriers are found at high frequencies in African, Asian, and Mediterranean populations and many affected children are born from parents who are both healthy carriers. The pathology is caused by the polymerization of the anomalous hemoglobin tetramers (HbS), that enclose a single amino acid substitution (Glu→Val) at cd6 of the *β*-globin chains, induced by a GAG→GTG mutation on the *β*-globin gene. 

Polymerization takes place in the postcapillary veins and can occur in less than one second, when the hemoglobin tetramers, having released their load of four atoms of oxygen, acquire their deoxy shape. The growing polymer fibers deform the round and elastic red cells into sickle shaped rigid erythrocytes that block the blood circulation. Sickle cells trapped in the spleen may induce massive hemolysis, chronic and acute infarctions in other organs, and in the skeleton inducing excruciating pain (crises), with progressive organ and tissues damage. The severe disease is leading to early disability and premature death, treatment is only supportive, cure might occur only in case of successful bone marrow transplantation and primary prevention is offered to couples at risk in many countries [1, 2].

## 2. Pathology and Genetic Background

The carriers of the trait (SCT), who are heterozygous for the A→T mutation, have approximately 40–50% HbS (*α*2/*β* ^S^2) in all their red cells, and usually show no symptoms because the normal HbA (*α*2/*β* ^A^2) coded by the normal allele inhibits the polymerization process in the red cells. Nevertheless, heterozygous may experience a milder transient pathology during triggering conditions [3]. 

The patients (homozygous or compound heterozygous) have inherited from both parents either the HbS gene or a combination of the HbS heterozygosity with *β*-thalassemia, HbC, HbE, HbD^Punjab^, HbO^Arab^, or other less common traits, the combination of which allows polymerization of the Hb tetramers in deoxy conditions. 

The severity of SCD can be quite variable and may express with very severe frequent crises and (fatal) complications or with only occasional crises and limited organ and tissue damage.

## 3. Modulating Factors: Genetic, External and Therapy Related

The severity of the SCD phenotype depends on genetic, external, and therapy-related factors. Among the genetic modulating factors, the expression of fetal hemoglobin (HbF) during post natal life is the most relevant. At birth, under normal conditions, HbF (*γ*2/*α*2) is present in the red cells at an average concentration of about 80% and it normally disappears (below 1%) during the first two years of life. This is the reason why newborn with SCD are perfectly healthy up to about 6 months of age, when the expression of the fetal hemoglobin has disappeared. While in the normal individuals HbF is replaced by the normal postnatal HbA, in SCD the HbF expression will be replaced by the abnormal HbS. In the healthy carrier both HbA and HbS will be present in an average ratio of 60 : 40.

In many cases of hereditary or acquired chronic anemia, the HbF concentration may remain higher than normal after birth. In SCD patients a moderate increase of 5–10% HbF may already reduce the symptoms of SCD considerably because HbF has a higher oxygen affinity, keeps some oxygen in the red cell in the postcapillary veins and inhibits HbS polymerization. For this reason, hydroxy urea (HU) treatment, a pharmacological enhancer of HbF expression, is largely used in preventing the symptoms of SCD [4] and also in *β*-thalassemia [5]. Not all these patients will equally respond to HU treatment because the natural and pharmacological expression of HbF in postnatal life is genetically determined [6]. The main determinant for a positive response is the presence of a particular polymorphism (Xmn-I) on the beta gene haplotype [5]. This C→T transition on the promoter region of the G*γ* gene (the main contributor to HbF expression normally silenced in adult life) is perhaps the most striking example of pharmacogenomic specificity. Moreover, HU protects from HbS polymerization because of the antiadherence effect on the red cells to the wall of the postcapillary veins [7].

A second important genetic factor modulating the severity of SCD is the coinheritance of *α*-thalassemia, another common trait in human, reducing the expression in *α*-globin chains, the non-*β* counterpart of both the fetal and postnatal tetramers, including HbS. 

Many carriers of SCT are also carriers of at least a single *α* ^+^-thalassemia gene (−*α*/*αα*) or are often homozygous (−*α*/−*α*) for these traits, presenting with hypochromic (MCH↓) and eventually microcytic (MCV↓) parameters and a moderate anemia. In fact, the majority of the healthy HbS carriers are identified “by accident”, after a patient controlled by routine (HPLC) laboratory analysis because of “microcytic anemia” not responding to iron therapy. The modulating effect of *α*-thalassemia has been long debated [8]. However, the many cases from our diagnostic service and the literature show a classic reduction of the HbS% in the carrier, indicating that *α*-globin, when scarce, preferentially bind to the normal *β* ^A^ rather than to the mutated *β* ^S^ globin chains [9]. This alternative is of course not present in the homozygous that has no *β* ^A^ chains to bind with *α* chains, but other factors related to *α*-thalassemia might have a modulating influence, perhaps the preferential binding of *α* chains for the normal *γ* chains (if present) rather than for *β* ^S^, perhaps the reduction in MCH, diminishing the stechiometric concentration of HbS in the red cells and herewith the speed and the chance of HbS to polymerize, perhaps the reduced size of the red cells (MCV↓) allowing a faster rheology, diminishing the critical flow-through time and the chance of HbS polymerization in the post capillary veins. 

External and treatment-related modulating factor play also a role in the course of the disease. Among the external one, dehydration, temperature, oxygen tension, and infections are the most important and will not be discussed in this paper.

Besides the above mentioned response to HU therapy, the treatment-related modulating factors are those related to transfusion dependency and iron overload. The golden rule in SCD treatment is to avoid blood transfusions as much as possible using eventually exchange transfusion which does not contribute to iron overload. Nevertheless, SCD patients who are often hospitalized for severe hemolytic crises are transfused and iron overloaded while some anemic SCD patients might even get iron prescriptions. Therefore, most SCD patients will have iron accumulation while iron depletion is only rarely observed generally in patients who (perhaps also thanks to that) have a mild phenotype. We believe that iron depletion could be a modulating factor which might contribute to ameliorate the phenotype of SCD and that a large evidence based study could be taken into consideration to validate our assumption illustrated by the following cases.

## 4. Case 1

We reported back in 2000 a case of HbS/*β*°-Thalassemia with a very mild pathology [10]. It concerned a mature woman mother of two, diagnosed with SCD at age 24, presenting at age 50 with the first hemolytic crisis during an infectious event (pneumonia). Patient showed a complete spleen atrophy, her HbF was 7%, and she was also homozygous for the most common *α* ^+^-thalassemia form, the −*α* ^3.7^ deletion defect (−*α*/−*α*). Due to the combined *β* and *α* Thalassemia, her Hb level was 9.8 g/dL and her MCV and MCH levels were typically reduced. Patient had never experienced an acute crisis, had never received a blood transfusion, and her ferritin level was low normal.

Case 1 tells us that the SCD condition in this severe HbS/*β*°-Thalassemia genotype was probably kept mild by (a) 7% HbF expression and (b) the presence of *α*-thalassemia and of never treated iron depletion, not even during the two iron requiring pregnancies, keeping this patients at low Hb, MCV, MCH, and ferritin levels for all her life.

## 5. Case 2

We have recently observed a 56 old female from Caribbean origin presenting with 10.6 g/dL Hb and a very pronounced microcytic hypochromic anemia. The Hb pattern was anomalous on HPLC, showing beside HbA a fraction corresponding to HbS but representing only 10% of the total. The patient had no HbF expression and an extreme iron depletion (ZPP 700 *μ*mol/mol in absence of led intoxication) and was carrier of the same −*α* ^3.7^ deletion as in case 1 (−*α*/*αα*). The very low expression of the abnormal fraction indicated an *α* rather than a *β*-gene mutation, but DNA sequence of the alpha genes was normal while sequence of the beta globin genes revealed the HbS mutation. A percentage of 10% HbS was inexplicable even in the presence of *α*-Thalassemia and after iron suppletion, the HbS concentration raised rapidly to 35%, the level expected in the HbS heterozygous in the presence of *α* ^+^-thalassemia heterozygosity. 

The anomalous Hb expression in this anemic HbS carrier tells us a few interesting things, namely that (a) the 10% HbS expression was not caused by the well-known preferential affinity for the formation of *α*/*β* ^A^ rather than *α*/*β* ^S^ dimers in the presence of the (−*α*/*αα*) genotype, and that (b) the HbS expression increased to the expected level of 35% after iron suppletion. This means that under conditions of iron depletion, the heme tends to be imbedded into the normal *β* ^A^rather than into the abnormal *β* ^S^ chains. These could also mean that in SCD and in condition of iron depletion, the expression of HbS could be inhibited in favor of the normal HbF. This cannot be demonstrated in this patients who, being heterozygous and in absence of the XmnI polymorphism, had no elevated HbF expression before or after iron therapy.

## 6. Discussion

The point to be further investigated is: could iron depletion, which induces low MCV and MCH just like *α*-thalassemia, have a positive modulating effect on the severity of SCD also because of a reduction of HbS expression in favor of an increased HbF? 

The iron housekeeping in our body is characterized by an efficient recycling and under normal conditions the main iron storage consists of the iron atoms built into the hemoglobin molecules. Each Hb tetramers contains 4 atoms harbored in the center of the 4 prosthetic groups and under normal conditions “heme iron” is fully recycled, while intake versus loss through the guts (±2 mg a day) is in slightly positive balance. 

The most significant nondisease-related changes in iron stores takes place during pregnancy (loss of 1000 mg) and during blood transfusion (gain of 200 mg fully absorbed from one bag of packed red cells). 

A significant increase in iron absorption is known to occur in thalassemia carriers. Therefore, treating the moderate anemia of these patients with iron therapy without checking the ferritin levels is useless and could be deleterious. 

The beta thalassemia major patient is transfusion dependent and secondary toxic overload is one of the major complications leading to premature death from cardiac disease. However, recent developments combining different chelating agents have led to a considerable health gain and life expectations [11].

In SCD treatment blood transfusion should be avoided as much as possible to limit iron accumulation. When blood transfusions are inevitable, the PCV value should not become too elevated, not to reduce the rheology in the postcapillary veins. Exchange transfusions without iron accumulation should be preferred when possible and be applied as a prophylactic measure to reduce crises frequency in severe cases. Unfortunately, being the ferritin threshold for chelation therapy usually 1000 *μ*g/L, SCD patients below this level are usually not chelated and live in a chronic state of iron overload. 

These authors do not need to underline the problems related to iron overload but would like to draw attention to the cases illustrated above, which indicate that iron depletion could be a possible modulating factor in reducing the severity of the pathology of SCD and that the usually accepted iron overload (up to 1000 *μ*g/L) could be an aggravating factor that may favor HbS polymerization. 

Iron depletion is not often observed in SCD. Nevertheless, based on the facts presented in our two cases, we have reasons to believe that keeping a SCD patient, especially if treated with hydroxyurea, at a low ferritin level by avoiding transfusions and/or by applying chelation therapy, might reduce the pathology of the disease by (a) favoring the formation of HbF rather than HbS tetramers, (b) by improving the rheology (low MCV), and reducing the molecular contacts in the red cell (low MCH) and herewith the chance of HbS polymerization and infarctions. 

We suggest that it might be worthwhile to monitor the clinical course of the disease in patients with and without iron depletion, with or without hydroxyurea treatment, and with the same SCD genotype.
